# Safety and efficacy of Rapamune® (Sirolimus) in kidney transplant recipients: results of a prospective post-marketing surveillance study in Korea

**DOI:** 10.1186/s12882-018-1002-6

**Published:** 2018-08-13

**Authors:** Hee Jung Jeon, Hahn-Ey Lee, Jaeseok Yang

**Affiliations:** 1 Department of Internal Medicine, Hallym University College of Medicine, 150 Seongan-ro, Gangdong-gu, Seoul, 134-701 South Korea; 2 Pfizer Pharmaceuticals Korea Ltd., Pfizer Tower 110, Toegye-ro, Jung-gu, Seoul, 100-771 South Korea; 3 Seoul National University College of Medicine Graduate School, 101 Daehak-ro, Jongno-gu, Seoul, 110-744 South Korea; 4 Transplantation Center, Department of Surgery, Seoul National University Hospital, Transplantation Research Institute, Seoul National University College of Medicine, 101 Daehak-ro, Jongno-gu, Seoul, 110-744 Republic of Korea

**Keywords:** Kidney transplantation, Post-marketing surveillance, Sirolimus

## Abstract

**Background:**

Few post-marketing surveillance studies have examined the safety and efficacy of Rapamune® (Sirolimus) in Asian countries. This study aimed to better understand safety and efficacy of Rapamune for kidney transplant recipients in the routine clinical practice setting in Korea.

**Methods:**

This was an open-label, non-comparative, observational, prospective, multi-center, post-marketing surveillance study conducted at 15 Korean transplant centers between 31 August 2009 and 24 September 2015. The subjects were administered Rapamune as part of routine practice. The safety was monitored based on reporting of adverse events (AEs). Efficacy endpoints included acute rejection, graft function, graft survival, and patient survival.

**Results:**

Rapamune was most commonly used for late conversion therapy after post-transplant 1 year and was substituted for anti-metabolites (63.6%) or calcineurin inhibitors (28.7%). The median treatment duration of Rapamune was 182 days. Among 209 subjects enrolled, AEs and adverse drug reactions (ADRs) were reported in 54.07% and 43.06% of subjects, respectively, in the safety analysis set. Most of the AEs were expected (96.21%), mild (75.83%), did not result in any action taken with regard to the study drug (72.99%), and resolved by the end of the study (75.36%). The most frequently reported AEs/ADRs were pharyngitis and diarrhea. Most of the serious AEs/ADRs occurred in one or two subjects. Unexpected ADRs of renal artery occlusion and cholangitis were reported by one subject each. The incidence of biopsy-proven acute rejection was 2.87%. At the end of the study, 99.51% of the subjects and their grafts had survived. The mean eGFR was 64.72 ± 19.56 mL/min.

**Conclusions:**

Rapamune had an acceptable safety profile in prevention of kidney allograft rejection in Korea.

## Background

Rapamune® (Sirolimus) is a mammalian target of rapamycin inhibitor. It is used as an immunosuppressive agent and is effective in preventing acute rejection and in preserving renal function in kidney transplant recipients [1, 2]. Rapamune is also used in either conversion or de novo regimens [3, 4]. Rapamune administered at a dose of 1 mg was approved for prophylaxis of organ rejection on 25 March 2006 in Korea. As required for any new medicine approved by the Ministry of Food and Drug Safety (MFDS; previously the Korea Food and Drug Administration), safety and efficacy information of the medicine needed to be provided in the routine practice setting during the re-examination period of 6 years from the approval date (25 March 2006 to 24 March 2012) and the extended period (25 March 2012 to 24 September 2015). Two mg of Rapamune was approved on 31 August 2009. Its safety and efficacy information needed to be provided during the remaining re-examination period of the 1 mg Rapamune dose.

This non-interventional, prospective, post-marketing surveillance study was designed to meet the requirement of the MFDS. Otherwise, there were no benefit-risk issues, safety concerns, or risk management measures that led to the initiation or imposition of this study. Background information on Rapamune can be obtained from the current version of the local product document (i.e., product label approved by the MFDS), which is the single reference safety document for information relating to Rapamune in this study.

Post-marketing surveillance is required to re-evaluate risk and benefit issues associated with particular drugs in the post-marketing environment because the safety and efficacy of drugs in a clinical trial setting may not coincide with those in clinical practice. The objective of the present study was to analyze post-marketing surveillance information to better understand safety and efficacy of Rapamune for kidney transplant recipients in the setting of routine clinical practice in Korea.

## Methods

### Study design and subjects

This was an open-label, non-comparative, observational, prospective, multi-center study conducted at 15 centers between 31 August 2009 and 24 September 2015. The subjects were administered Rapamune as part of routine practice at Korean transplantation centers by accredited physicians or surgeons. The use and dosage recommendations for Rapamune were based on the approved local product document and were adjusted solely according to medical and therapeutic necessity. Observation of subjects was performed for 6 ± 1 months after initiating Rapamune administration or until completion of Rapamune administration, whichever was earlier. There were no mandatory visits or activities during the study. The investigator recorded the following information on the subject’s case report form: demographic information, subject status, transplantation status, general medical history, prior immunosuppressive medication, administration of Rapamune, concomitant medications and therapies, trough level of immunosuppressive medications, and safety and efficacy information. Past medical history was defined as any medical illness that had been cured in the past and did not persist at the time of study enrollment.

To be eligible for enrollment into the study, subjects needed to be ≥13 years of age, renal transplant recipients, newly administered Rapamune after a contract was made between Pfizer Korea and an investigator and/or an institution for conducting this study, and to have provided personally signed and dated informed consent document acknowledging having been informed of all pertinent aspects of the study. For patients aged less than 19 years, informed consent was obtained from both patient and parents or guardians. Subjects presenting with any of the following were excluded from the study: (1) any subjects who did not agree with usage of his/her information; (2) subjects with known hypersensitivity to Rapamune or its derivatives, or any excipients in the formulation; (3) subjects with hereditary problems such as galactose intolerance, Lapp lactase deficiency, or glucose-galactose malabsorption because Rapamune contains lactose; and (4) any reproductive aged woman with a birth plan.

This study was approved by the Institutional Review Board of each participating center, and it was performed in accordance with the Helsinki Declaration of 2000 and the Declaration of Istanbul 2008.

### Outcome measurement

Safety was monitored throughout the observation period. An adverse event (AE) was defined as any untoward medical occurrence including symptom, sign, and disease in a subject administered Rapamune. The event did not need to have a causal relationship with the product or usage. AE recording included identification of the AE, onset and resolution dates, severity, action taken with Rapamune, seriousness, outcome, and causality assessment (certain, probable/likely, possible, unlikely, conditional/unclassified, unassessable/unclassifiable). For all AEs, sufficient information should have been obtained by the investigator to determine the causality of each AE. For AEs with a causal relationship to Rapamune, follow-up by the investigator was required until the event or its sequelae resolved or stabilized at a level acceptable to the investigator. The investigators decided the medical term of AEs clinically, and all AEs were standardized and classified based on systemic organ class and preferred term according to the World Health Organization Adverse Reaction Terminology, version 092. A serious AE (SAE) was defined as any untoward medical occurrence in a subject administered a medicinal product any dose that: (1) resulted in death, (2) was life-threatening, (3) required inpatient hospitalization or prolongation of hospitalization, (4) resulted in persistent or significant disability/incapacity, or (5) resulted in congenital anomaly or birth defect. All AEs, except for those for which a causal relationship to the study drug was “unlikely”, were considered as adverse drug reactions (ADRs) in this study.

The efficacy endpoints included incidence rate of biopsy-confirmed acute rejection using Banff 09 diagnostic categories for renal allograft biopsies, graft function, patient survival, and graft survival. Graft function was evaluated by estimated glomerular filtration rate (eGFR) using the Nankivell formula calculated with the following equation: GFR (mL/min) = 6.7/creatinine (mmol/L) – urea (mmol/L)/2 + body weight (kg)/4–100/height (m)^2^ + (35 [male] or 25 [female]). The Nankivell equation is one of the most accurate methods of the creatinine-based estimates for eGFR in kidney transplant recipients [5, 6]. Graft loss was defined as the initiation of dialysis, graft nephrectomy, re-transplantation, or patient death with a functioning graft.

### Sample size determination

No formal sample size calculation was performed in this observational study. At least 600 subjects were to be enrolled in this study based on the requirement of the MFDS. However, considering the difficulty of subject enrollment, the MFDS extended the re-examination period and adjusted the number of subjects. Therefore, the planned enrollment was at least 200 subjects during the re-examination period and the extended period in this study.

### Statistical analyses

Descriptive summary statistics for continuous variables included number of subjects, mean, median, standard deviation, minimum, and maximum. Descriptive statistics for categorical variables were given as frequencies and percentages. The proportion of subjects who experienced AEs were estimated and compared among subcategories of each baseline and treatment characteristic using the chi-square test or Fisher’s exact test. The proportion of subjects who had survived and whose graft had survived at the time of final evaluation was calculated with its 95% confidence interval estimated for analysis of patient and graft survival. All statistical analyses were carried out with statistical analysis system (SAS) software versions 9.3 and 9.4. All test statistics were based on 2-sided tests with the statistical significance level of 0.05.

## Results

### Patient demographics and baseline characteristics

A total of 209 kidney transplant recipients who were treated with Rapamune were enrolled. All subjects were included in both the safety analysis set and the efficacy analysis set. The baseline demographics and clinical characteristics of subjects in this study are summarized in Table 1. The mean age was 47.93 ± 13.10 years and 61.24% of the subjects were male. The majority of subjects (87.56%) were under 65 years of age. The most common cause of primary kidney failure was hypertension followed by glomerulonephritis, diabetes mellitus, and polycystic kidney disease. Median number of HLA mismatch was 3.00 (range: 0 to 6). Among 208 subjects who had type of donor data, 132 (63.46%) subjects underwent living donor kidney transplantation, and the mean age of donors was 41.49 ± 13.16 years. Mean positive class I and class II panel reactive antibodies were 6.35 ± 21.50% and 5.89 ± 17.39%, respectively (Table 1).

**Table 1:** Clinical characteristics of study subjects

	Total (*N* = 209)
Age, years	47.93 ± 13.10
Age group, n (%)	
< 65 years	183 (87.56)
≥ 65 years	26 (12.44)
Sex, male, n (%)	128 (61.24)
Height, cm	164.85 ± 8.80
Body Weight, kg	61.73 ± 10.75
Allergy history, n (%)	5 (2.39)
Secondary transplantation, n (%)	10 (4.78%)
Primary cause of kidney failure, n (%)	
Hypertension	56 (26.79)
Diabetes mellitus	33 (15.79)
Glomerulonephritis	55 (26.32)
Polycystic kidney disease	11 (5.26)
Others	54 (25.84)
Panel reactive antibody of recipients (%)	
Class I	6.35 ± 21.50
Class II	5.89 ± 17.39
Type of donation, n (%)	
Living donor	132 (63.46)
Deceased donor	76 (36.54)
Age of donors, years	41.49 ± 13.16
Past general medical history, n (%)^a^	141 (67.46%)
Liver disease, n (%)	13 (6.22%)
Concomitant immunosuppressive medication, n (%)^b^	
Monotherapy	44 (21.05)
Dual therapy	140 (66.99)
Triple therapy	25 (11.96)
Trough level of cyclosporine, ng/mL	
Immunoassay	72.84 ± 47.01
HPLC method	100.18 ± 64.80
Trough level of tacrolimus, ng/mL	
Immunoassay	4.34 ± 1.77
HPLC method	4.51 ± 3.25
Trough level of mycophenolic acid, ng/mL	
HPLC method	1.53 ± 0.86
Time between transplantation and the first administration of Rapamune, days, median (range)	median 909 (range: 9 to 10,816)
< 6 months, n (%)	28 (13.40)
6 months to < 1 year, n (%)	20 (9.57)
1 year to < 5 years, n (%)	94 (44.98)
≥ 5 years, n (%)	65 (31.10)
Total administration period of Rapamune, days	median 182 (range: 4 to 971)
< 6 months, n (%)	96 (45.93)
6 month to < 1 year, n (%)	99 (47.37)
≥ 1 year, n (%)	14 (6.70)
Daily dose of Rapamune, mg	1.79 ± 0.68
< 2 mg, n (%)	112 (53.59)
2 mg, n (%)	46 (22.01)
> 2 mg, n (%)	51 (24.40)
Trough level of Rapamune, ng/mL	
Immunoassay	5.45 ± 2.59
HPLC method	7.87 ± 4.24

Most numerical values are presented as a mean ± standard deviation, and categorical values are expressed as a frequency (percentage)

*Abbreviations: cm* centimeter(s), *kg* kilogram(s), *HPLC* high performance liquid chromatography

^a^Past general medical history was defined as any medical illness that had been cured in the past and did not persist at the time of study enrollment

^b^Monotherapy: steroid only, calcineurin inhibitor only, or anti-metabolite only; Dual therapy: steroid plus calcineurin inhibitor, steroid plus anti-metabolite, or calcineurin inhibitor plus anti-metabolite; Triple therapy: steroid plus calcineurin inhibitor plus anti-metabolite

### Immunosuppressive medications

All 209 subjects took concomitant immunosuppressive medications during the study, and most subjects (78.95%) took two or more concomitant immunosuppressive medications. The most common concomitant immunosuppressants were a dual combination of calcineurin inhibitor and steroid (41.15%). Rapamune was substituted for other immunosuppressants (85.4%) or was added on other immunosuppressants (14.6%). Among the conversion group, anti-metabolites were converted to Rapamune most frequently (63.6%) and calcineurin inhibitors were converted to Rapamune in 28.7%.

The median time from kidney transplantation to the first administration of Rapamune was 909 days (range: 9 to 10,816 days). Rapamune was introduced in 28 subjects (13.40%) within 6 months; however, most subjects (159 subjects, 76.08%) began to take Rapamune 1 year after transplantation (Table 1). Calcineurin inhibitors were also converted to Rapamune within 6 months only in 13.51% and conversion occurred after 1 year in most cases (75.7%).

The median administration period of Rapamune was 182 days (range: 4 to 971 days). The mean daily dose of Rapamune is 1.79 ± 0.68 mg/day, and more than half of the subjects (53.59%) were treated with daily dose of < 2 mg/day. The mean trough level of Rapamune was 5.45 ± 2.59 ng/mL as measured using immunoassay and 7.87 ± 4.24 ng/mL as determined using high performance liquid chromatography (HPLC). The mean trough level of concomitant tacrolimus was 4.34 ± 1.77 ng/mL and 4.51 ± 3.25 ng/mL as determined using immunoassay and HPLC, respectively. The mean trough level of concomitant cyclosporine was 72.84 ± 47.01 ng/mL and 100.18 ± 64.80 ng/mL in the same respective order (Table 1).

### Safety

At the time of study completion, 3 subjects were lost to follow up in our study, and 206 subjects were evaluated in the safety analysis set. Among 206 subjects, 167 (79.9%) were continuing Rapamune administration and 39 (18.66%) discontinued the treatment. Discontinuation was because of AEs in 87.18% (34/39) of the subjects and for other reasons in 10.26% (4/39). The other reasons included elevation of serum creatinine, subjective refusal, informed consent withdrawal, and cecal tuberculosis. Another subject discontinued due to AEs and was switched from Rapamune to mycophenolic acid. Four subjects restarted Rapamune, but 35 subjects permanently discontinued Rapamune due to AEs. The most commonly reported AEs that led to discontinuation were azotemia in 6 subjects and diarrhea in 5 subjects. The list of AEs that lead to discontinuation of Rapamune is summarized in Table 2.

**Table 2:** List of adverse events lead to discontinuation of Rapamune

Systemic organ class and preferred term^a^	Total (*N* = 209)	
	Number of Subjects (%)	Number of Adverse Events
Resistance mechanism disorders		
Infection susceptibility increased	1 (0.48)	1
Polyomavirus infection	1 (0.48)	1
Gastro-intestinal system disorders		
Diarrhoea	5 (2.39)	5
Stomatitis	3 (1.44)	3
Stomatitis ulcerative	1 (0.48)	1
Abdominal pain	2 (0.96)	2
Nausea	1 (0.48)	1
Urinary system disorders		
Azotaemia	6 (2.87)	6
Albuminuria	1 (0.48)	1
Oliguria	1 (0.48)	1
Skin and appendages disorders		
Acne	1 (0.48)	1
Rash	1 (0.48)	1
Rash erythematous	2 (0.96)	2
Dermatitis	1 (0.48)	1
Metabolic and nutritional disorders		
Diabetes mellitus	1 (0.48)	1
Diabetes mellitus aggravated	1 (0.48)	1
Plasma osmolality increased	1 (0.48)	1
Body as a whole– general disorders		
Edema peripheral	1 (0.48)	1
Face edema	2 (0.96)	2
Anaphylactic reaction	1 (0.48)	1
Liver and biliary system disorders		
Hepatic function abnormal	1 (0.48)	1
Cholangitis	1 (0.48)	1
Musculo-skeletal system disorders		
Avascular necrosis bone	1 (0.48)	1
Secondary terms – events		
Transplant rejection	2 (0.96)	2
Respiratory system disorders		
Coughing (≥ 4 weeks)	1 (0.48)	1
White cell and RES disorders		
Leucopenia	1 (0.48)	1

^a^Adverse drug reactions classified according to the World Health Organization Adverse Reaction Terminology (WHO-ART) version 092 coding dictionary

All AEs and ADRs that occurred during the study are summarized in Table 3. Overall, 113 (54.07%) subjects experienced 211 AEs. The majority of AEs were expected (96.21%), non-serious (86.73%), mild (75.83%), did not result in any action taken with regard to the study drug (72.99%), and resolved by the end of the study (75.36%). AEs occurred most frequently in resistance mechanism disorders (48, 22.97%) and gastro-intestinal system disorders (39, 18.66%). The most frequently reported AEs by preferred term were pharyngitis (24, 11.48%) and diarrhea (12, 5.74%). Almost all AEs were mild (75.83%, 160/211) or moderate (20.38%, 43/211). AEs of severe intensity were reported in 3.79% (8/211) of subjects.

**Table 3:** Adverse events and adverse drug reactions classified by systemic organ class according to the World Health Organization Adverse Reaction Terminology

Systemic organ class and preferred term^a^	Total (*N* = 209)			
	Adverse Event		Adverse Drug Reaction	
	Number of Subjects (%)	Number of Events	Number of Subjects (%)	Number of Events
Total	113 (54.07)	211	90 (43.06)	156
Resistance mechanism disorders	48 (22.97)	66	35 (16.75)	50
Pharyngitis	24 (11.48)	32	23 (11.00)	31
Upper respiratory tract infection	10 (4.78)	15	5 (2.39)	10
Urinary tract infection	4 (1.91)	4	1 (0.48)	1
Rhinitis	3 (1.44)	3	0 (0.00)	0
Pneumonia	3 (1.44)	3	2 (0.96)	2
Herpes simplex	2 (0.96)	2	2 (0.96)	2
Cystitis	2 (0.96)	2	1 (0.48)	1
Infection susceptibility increased	1 (0.48)	1	1 (0.48)	1
Abscess	1 (0.48)	1	0 (0.00)	0
Herpes zoster	1 (0.48)	1	1 (0.48)	1
Dermatitis fungal	1 (0.48)	1	1 (0.48)	1
Polyomavirus infection	1 (0.48)	1	0 (0.00)	0
Gastro-intestinal system disorders	39 (18.66)	49	34 (16.27)	38
Diarrhoea	12 (5.74)	13	11 (5.26)	11
Stomatitis	7 (3.35)	7	7 (3.35)	7
Stomatitis ulcerative	7 (3.35)	7	7 (3.35)	7
Abdominal pain	4 (1.91)	4	4 (1.91)	4
Gastritis	3 (1.44)	3	2 (0.96)	2
Constipation	2 (0.96)	2	1 (0.48)	1
Dyspepsia	2 (0.96)	3	2 (0.96)	3
Nausea	2 (0.96)	2	1 (0.48)	1
Enteritis	2 (0.96)	2	1 (0.48)	1
Vomiting	1 (0.48)	2	0 (0.00)	0
Ileus	1 (0.48)	2	0 (0.00)	0
Tooth caries	1 (0.48)	1	0 (0.00)	0
Periodontal destruction	1 (0.48)	1	1 (0.48)	1
Urinary system disorders	15 (7.18)	16	11 (5.26)	11
Azotaemia	10 (4.78)	10	7 (3.35)	7
Albuminuria	2 (0.96)	2	2 (0.96)	2
Nephropathy toxic	1 (0.48)	1	0 (0.00)	0
Urinary tract disorder	1 (0.48)	1	0 (0.00)	0
Oliguria	1 (0.48)	1	1 (0.48)	1
Renal artery occlusion	1 (0.48)	1	1 (0.48)	1
Skin and appendages disorders	15 (7.18)	17	13 (6.22)	14
Acne	5 (2.39)	5	5 (2.39)	5
Rash	4 (1.91)	4	4 (1.91)	4
Onychomycosis	2 (0.96)	2	1 (0.48)	1
Rash erythematous	2 (0.96)	2	2 (0.96)	2
Folliculitis	1 (0.48)	1	0 (0.00)	0
Eczema	1 (0.48)	1	1 (0.48)	1
Dermatitis contact	1 (0.48)	1	0 (0.00)	0
Dermatitis	1 (0.48)	1	1 (0.48)	1
Metabolic and nutritional disorders	13 (6.22)	14	11 (5.26)	12
Diabetes mellitus	3 (1.44)	3	3 (1.44)	3
Hyperlipemia	4 (1.91)	4	4 (1.91)	4
Hyperkalemia	2 (0.96)	2	2 (0.96)	2
Diabetes mellitus aggravated	2 (0.96)	2	2 (0.96)	2
Hyperphosphatemia	1 (0.48)	1	0 (0.00)	0
Glycosuria	1 (0.48)	1	1 (0.48)	1
Plasma osmolality increased	1 (0.48)	1	0 (0.00)	0
Body as a whole– general disorders	11 (5.26)	11	8 (3.83)	8
Edema peripheral	2 (0.96)	2	2 (0.96)	2
Face edema	2 (0.96)	2	2 (0.96)	2
Fever	2 (0.96)	2	1 (0.48)	1
Allergic reaction	1 (0.48)	1	1 (0.48)	1
Leg pain	1 (0.48)	1	0 (0.00)	0
Ascites	1 (0.48)	1	0 (0.00)	0
Anaphylactic reaction	1 (0.48)	1	1 (0.48)	1
Fatigue	1 (0.48)	1	1 (0.48)	1
Liver and biliary system disorders	6 (2.87)	7	3 (1.44)	4
SGOT increased	2 (0.96)	2	1 (0.48)	1
Hepatic enzymes increased	2 (0.96)	2	0 (0.00)	0
SGPT increased	1 (0.48)	1	1 (0.48)	1
Hepatic function abnormal	1 (0.48)	1	1 (0.48)	1
Cholangitis	1 (0.48)	1	1 (0.48)	1
Musculo-skeletal system disorders	6 (2.87)	6	4 (1.91)	4
Osteoporosis	2 (0.96)	2	2 (0.96)	2
Fracture	2 (0.96)	2	0 (0.00)	0
Myalgia	1 (0.48)	1	1 (0.48)	1
Avascular necrosis bone	1 (0.48)	1	1 (0.48)	1
Secondary terms – events	6 (2.87)	6	3 (1.44)	3
Transplant rejection	6 (2.87)	6	3 (1.44)	3
Respiratory system disorders	4 (1.91)	4	3 (1.44)	3
Coughing	3 (1.44)	3	3 (1.44)	3
Dysonoea	1 (0.48)	1	0 (0.00)	0
White cell and RES disorders	3 (1.44)	3	2 (0.96)	2
Leucopenia	2 (0.96)	2	2 (0.96)	2
Granulocyopenia	1 (0.48)	1	0 (0.00)	0
Central & peripheral nervous system disorders	3 (1.44)	4	2 (0.96)	3
Headache	2 (0.96)	2	1 (0.48)	1
Hypoaesthesia	1 (0.48)	1	1 (0.48)	1
Tremor	1 (0.48)	1	1 (0.48)	1
Red blood cell disorders	2 (0.96)	2	2 (0.96)	2
Anaemia	1 (0.48)	1	1 (0.48)	1
Polycythaemia	1 (0.48)	1	1 (0.48)	1
Endocrine disorders	1 (0.48)	1	0 (0.00)	0
Hyperparathyroidism	1 (0.48)	1	0 (0.00)	0
Vision disorders	1 (0.48)	1	1 (0.48)	1
Meibomianitis	1 (0.48)	1	1 (0.48)	1
Myo-, endo-, pericardial & valve disorders	1 (0.48)	1	0 (0.00)	0
Angina pectoris aggravated	1 (0.48)	1	0 (0.00)	0
Cardiovascular disorders, general	1 (0.48)	1	0 (0.00)	0
Hypertension	1 (0.48)	1	0 (0.00)	0
Psychiatric disorders	1 (0.48)	1	1 (0.48)	1
Insomnia	1 (0.48)	1	1 (0.48)	1
Platelet, bleeding & clotting disorders	1 (0.48)	1	0 (0.00)	0
Thrombocytopenia	1 (0.48)	1	0 (0.00)	0

A*bbreviations: RES* reticuloendothelial system

^a^Adverse drug reactions classified according to the World Health Organization Adverse Reaction Terminology (WHO-ART) version 092 coding dictionary

The proportion of subjects with AEs within each category of predefined baseline and clinical characteristic was compared using chi-square test or Fisher’s exact test. There were statistically significant differences in proportion of subjects with AEs across subgroups in the following categories. The subjects with the time between transplantation and first administration of Rapamune in less than 1 year had more AEs than those in 1 to 5 years or in longer than 5 years (70.21% vs. 48.42% vs. 52.31% respectively, *P* = 0.0445). More AEs were evident for subjects who had a past medical history compared to those without such history (60.28% vs. 41.18%, *P* = 0.0094), and subjects who had liver disease compared to those without liver disease (92.31% vs. 51.53%, *P* = 0.0043). AEs were significantly different according to the total administration duration of Rapamune, with rates of 69.79% for < 6 months, 45.45% for 6 months to 1 year, and 7.14% for ≥1 year (*P* < 0.0001). Subjects continuously administered Rapamune at the time of study completion had significantly less AEs than those who had discontinued Rapamune (44.31% vs. 92.31%, *P* < 0.0001).

Among 211 AEs from 113 subjects, 156 AEs (73.9%) from 90 subjects were considered as ADRs according to the causality assessment of the investigator (conditional/unclassified: 41.71%; possible: 21.33%; probable/likely: 4.74%; certain: 4.74%; unassessable/unclassifiable: 1.42%). ADRs occurred most frequently in the systemic organ class of resistance mechanism disorders (35, 16.75%) and gastro-intestinal system disorders (34, 16.27%). The most frequently reported ADRs by preferred term were pharyngitis (23, 11.00%) and diarrhea (11, 5.26%). Other ADRs that reported in ≥1% of subjects included stomatitis, stomatitis ulcerative, azotemia (each reported by 7 subjects, 3.35% each), upper respiratory tract infection, acne (each reported by 5 subjects, 2.39% each), abdominal pain, rash (each reported by 4 subjects, 1.91% each), diabetes mellitus, transplant rejection, and coughing (each reported by 3 subjects, 1.44% each). Seven (3.35%) subjects experienced 8 unexpected AEs during the study, of which 2 (0.96%) subjects experienced 2 unexpected ADRs (renal artery occlusion and cholangitis in 1 subject, 0.48% each). The one death involved a 78-year-old male subject who had a medical history of liver disease. He died on Study Day 61 due to a SAE (acute cholangitis), which was considered to be possibly related to the study drug by the investigator.

All SAEs/serious adverse drug reactions (SADRs) by World Health Organization Adverse Reaction Terminology systemic organ class and preferred term are summarized in Table 4 for the safety analysis set. A total of 16 subjects (7.66%) experienced 28 SAEs during the study. All SAEs were reported in 1 or 2 subjects except for azotemia, which was reported in 4 (1.91%) subjects. Six (2.87%) subjects experienced 9 SADRs, which were azotemia (3, 1.44%), diarrhea (2, 0.96%), pneumonia, increased infection susceptibility, herpes zoster, and cholangitis (each reported by 1 subject, 0.48% each). A total of 5 (2.39%) subjects reported 5 unexpected SAEs; 1 (0.48%) subject reported 1 unexpected SADR; the cholangitis was fatal.

**Table 4:** Serious adverse events and serious adverse drug reactions classified by systemic organ class according to the World Health Organization Adverse Reaction Terminology

Systemic organ class and preferred term^a^	Total (*N* = 209)			
	SAEs		SADRs	
	Number of Subjects (%)	Number of Events	Number of Subjects (%)	Number of Events
Total	16 (7.66)	28	6 (2.87)	9
Resistance mechanism disorders	6 (2.87)	7	3 (1.44)	3
Pneumonia	2 (0.96)	2	1 (0.48)	1
Cystitis	1 (0.48)	1	0 (0.00)	0
Infection susceptibility increased	1 (0.48)	1	1 (0.48)	1
Abscess	1 (0.48)	1	0 (0.00)	0
Herpes zoster	1 (0.48)	1	1 (0.48)	1
Polyomavirus infection	1 (0.48)	1	0 (0.00)	0
Gastro-intestinal system disorders	3 (1.44)	4	2 (0.96)	2
Diarrhea	2 (0.96)	2	2 (0.96)	2
Ileus	1 (0.48)	2	0 (0.00)	0
Urinary system disorders	5 (2.39)	5	3 (1.44)	3
Azotemia	4 (1.91)	4	3 (1.44)	3
Urinary tract disorder	1 (0.48)	1	0 (0.00)	0
Skin and appendages disorders	1 (0.48)	1	0 (0.00)	0
Dermatitis contact	1 (0.48)	1	0 (0.00)	0
Metabolic and nutritional disorders	1 (0.48)	1	0 (0.00)	0
Plasma osmolality increased	1 (0.48)	1	0 (0.00)	0
Body as a whole– general disorders	1 (0.48)	1	0 (0.00)	0
Ascites	1 (0.48)	1	0 (0.00)	0
Liver and biliary system disorders	1 (0.48)	1	1 (0.48)	1
Cholangitis	1 (0.48)	1	1 (0.48)	1
Musculo-skeletal system disorders	1 (0.48)	1	0 (0.00)	0
Fracture	1 (0.48)	1	0 (0.00)	0
Secondary terms – events	2 (0.96)	2	0 (0.00)	0
Transplant rejection	2 (0.96)	2	0 (0.00)	0
Respiratory system disorders	1 (0.48)	1	0 (0.00)	0
Dyspnea	1 (0.48)	1	0 (0.00)	0
White cell and RES disorders	1 (0.48)	1	0 (0.00)	0
Granulocyopenia	1 (0.48)	1	0 (0.00)	0
Endocrine disorders	1 (0.48)	1	0 (0.00)	0
Hyperparathyroidism	1 (0.48)	1	0 (0.00)	0
Myo-, endo-, pericardial & valve disorders	1 (0.48)	1	0 (0.00)	0
Angina pectoris aggravated	1 (0.48)	1	0 (0.00)	0
Cardiovascular disorders, general	1 (0.48)	1	0 (0.00)	0
Hypertension	1 (0.48)	1	0 (0.00)	0

*Abbreviations: RES* reticuloendothelial system, *SAEs* serious adverse events, *SADRs* serious adverse drug reactions

^a^Adverse drug reactions classified according to the World Health Organization Adverse Reaction Terminology (WHO-ART) version 092 coding dictionary

### Efficacy

Efficacy data for biopsy-confirmed acute rejection, patient survival, graft survival, and graft function evaluated by eGFR are summarized in Table 5. In the efficacy analysis set, 6 subjects had biopsy-confirmed acute rejection using Banff 09 diagnostic categories for renal allograft biopsies. The incidence of acute rejection was 2.87%. None of 6 subjects who were diagnosed as acute rejection had donor specific anti-HLA antibodies, and either acute or chronic antibody-mediated rejection was not found in their biopsy results. Among 6 subjects who had acute rejection episodes, 5 patients had begun to take Rapamune within the first year after transplantation. In their biopsy results, 3 subjects had borderline change, 2 subjects had acute T cell mediated rejection type IA, and 1 subject had acute T cell mediated rejection type IB. Among them, 4 subjects received steroid pulse therapy except 2 subjects who had borderline change. Five subjects recovered their graft function, however, graft failure occurred in 1 subject who had acute T cell mediated rejection type IB after study completion. Among 206 subjects who had patient survival data, 205 subjects survived at the time of final evaluation. The survival rate was 99.51%. Among 206 subjects who had graft survival data, 205 subjects had no graft loss at the time of final evaluation. The proportion of subjects with a surviving graft was 99.51% at a median follow-up of 182 days. The median and mean eGFR calculated by Nankivell formula was 67.07 mL/min (range: 4.80 to 113.27 mL/min) and 64.72 ± 19.56 mL/min, respectively for the 182 subjects who had graft function data in the efficacy analysis set.

**Table 5:** Efficacy analysis

	Total (*N* = 209)		
	Yes, n (%)	95% CI (lower, upper)	Total, n (%)
Acute rejection	6 (2.87)	0.91, 5.79	209 (100.00)
Patient survival^a^	205 (99.51)	98.57, 100.00	206 (98.56)
Graft survival^a^	205 (99.51)	98.57, 100.00	206 (98.56)
Graft function^b^			
eGFR (mL/min)	64.72 ± 19.56^c^		182 (87.08)

*Abbreviations: CI* confidence interval, *eGFR* estimated glomerular filtration rate

^a^Data were not available for 3 subjects due to loss of follow-up

^b^Data were not available for 27 subjects

^c^eGFR is expressed as a mean ± standard deviation

## Discussion

Post-marketing surveillance studies can reveal hitherto unknown or unexpected safety issues, providing clinicians with valuable information about the drugs. The present study was a non-interventional study of the safety and efficacy of Rapamune used in routine clinical practice in Korean kidney transplant recipients. Rapamune was most commonly used for late conversion therapy after post-transplant 1 year and was substituted for anti-metabolites (63.6%) or calcineurin inhibitors (28.7%). Most of the AEs were expected, mild, and self-limiting. Furthermore, these AEs might not be attributed to Rapamune as there was no control group. Kidney allograft rejection rate was low with good renal function and graft survival at short-term follow-up. Overall, we demonstrated the acceptable tolerability of Rapamune in Korean kidney transplant recipients in routine clinical environment.

Importantly, the cumulative incidence of AEs (54.07%) was lower than that reported in previous randomized controlled trials [7–9]. The previous studies reported that almost all subjects treated with sirolimus had at least one AE [7–9]. Furthermore, 16.75% of subjects permanently discontinued Rapamune due to AEs in this study, whereas AEs led to discontinuation of study drug in 21–23% of subjects in the clinical trials [8, 9]. These differences between clinical trials and post-marketing surveillance study could be explained by the more stringent protocols of randomized controlled trials. In this observational, non-interventional study, clinicians could select the optimal dose of Rapamune according to the response of each patient in terms of the balance between benefit and risk. However, in the clinical trials, study drug dose and method of administration was determined according to the strict protocol of the trials. Euvrard et al. [9] reported that subjects who were converted to sirolimus with rapid protocols had a higher rate of discontinuation as well as a higher incidence of SAEs than those with progressive protocols. While the target trough level of sirolimus in the clinical trials were 5–15 ng/mL, the mean trough level reported here was 5.45 ± 2.59 ng/mL and most of the subjects had daily dose of Rapamune of 2 mg (22.01%) or less than 2 mg (53.59%) in the present study. Lower trough concentrations with gradual dose adjustment in the real clinical practice could have resulted in better safety profile in the present study.

Presently, the most common AEs were resistance mechanism disorders including pharyngitis and gastrointestinal system disorders such as diarrhea. These findings were consistent with previous studies [7–9]. However, most studies commonly reported acne, dyslipidemia, mouth ulceration, peripheral edema, and proteinuria with sirolimus use [7–9]. Relatively few subjects experienced these AEs in the present study, and this might be also explained by the aforementioned reasons. Additionally, our study showed that the subjects with the time between transplantation and first administration of Rapamune in less than 1 year had significantly more AEs than those in longer than 1 year. Obviously, most AEs in the Rapamune use are concentration-related. Doses and levels obtained during the first year after transplantation may differ from those in longer than 1 year could have contributed to the significantly different incidence of AEs between the two groups.

At the end of the study, patient survival and graft survival rates were 99.51%. Graft failure occurred in 1 case and the cause of graft failure was death with a functioning graft from cholangitis. The incidence of biopsy-proven acute rejection was 2.87% and eGFR (mean 64.72 ± 19.56 mL/min) was remained stable in this study. These results are consistent with previous studies that investigated the efficacy of sirolimus [1, 2, 8, 10, 11]. Even though treatment duration of Rapamune in this study was relatively short (median treatment duration: 182 days; range: 4 to 971 days), these findings indicated that Rapamune was effective in prevention of kidney allograft rejection and in preserving renal function especially in kidney transplant subjects who had begun to take Rapamune 1 year after transplantation.

The present study has some limitations. First, this study was an observational post-marketing surveillance study to satisfy the requirements of the MFDS. The protocol and statistical analysis plan were determined by regulation of the MFDS rather than characteristics of specific disease or drug. The non-interventional, non-comparative, and non-randomized design of this study is an intrinsic limitation. However, the observational design of this study is more likely to provide information related to routine clinical practice compared to randomized controlled trials. Second, the treatment duration of Rapamune was relatively short (median: 182 days), and the time from kidney transplantation to the first dose of Rapamune was heterogenous (median: 909 days; range: 9 to 10,816 days). However, most AEs occurred in the early period after initiation of Rapamune, and the majority of subjects (77.29%) received the first dose of Rapamune later than 1 year after kidney transplantation. Third, the study population was relatively small. Large-scale, long-term studies would be helpful. Nevertheless, the present study is meaningful as it provides important information about safety and efficacy of Rapamune in routine clinical practice for Asian kidney transplant subjects; most of the prior studies have been performed with Caucasians.

## Conclusions

Rapamune had an acceptable safety profile in prevention of kidney allograft rejection in Korea. The study results had no impact on the known benefit-risk balance of Rapamune.

## Abbreviations

ADR: Adverse drug reaction
AE: Adverse event
eGFR: Estimated glomerular filtration rate
MFDS: The Ministry of Food and Drug Safety
SADR: Serious adverse drug reaction
SAE: Serious adverse event
